# Silencing of Viral Elements: An Available Cure for Schizophrenia?

**DOI:** 10.3389/fpsyt.2017.00284

**Published:** 2017-12-13

**Authors:** Hans C. Klein

**Affiliations:** ^1^Department of Psychiatry and Medical Imaging Centre, University of Groningen, Groningen, Netherlands

**Keywords:** neurogenesis, HSV-1, hippocampus, schizophrenia, epigenetics mechanisms of plasticity

## The Problem

Scientific evidence for various infectious agents as cause for psychosis in schizophrenia is not robust. Many pathogens that influence brain development might play a role in disease causation. It has been shown that influenza exposure before birth (1), exposure to herpes simplex during birth (2), or exposition to Toxoplasma gondii and other pathogens (3) increases risk for schizophrenia and/or compromises cognition. During psychosis, viral and also ancient retroviral elements become may activated in the brain (4, 5). The implication of this research for use in clinical practice is ambiguous, because a robust virus test is lacking. The current article explores the speculative, but testable hypothesis that HSV-1 a neurotropic virus infecting specific limbic brain regions is a necessary—but not sufficient—cause of psychosis in a significant proportion of schizophrenia patients. The test should qualify Koch’s adapted postulates for latent pathogens (6): It should be present in the affected organ only in diseased cases, it should explain the pathogenetic changes of the individual and last, the pathogen should be transmissible and carry a corresponding disease in an animal model. We chose to focus on HSV-1, which specifically infects limbic brain regions and explains neuropathological and behavioral deficits in acute psychotic, and further detioration in chronically schizophrenic patients, but may also accounts for extremely high (up to 60%) concordance in monozygotic twins (7). HSV-1 is indeed abundantly present; up to 80% of adults harbor the virus and it is the only known virus, which has specific neurotropism for the limbic regions of the brain (8) involved in the inflammatory pathophysiology of schizophrenia (9). Transmitting the HSV in animals yields pathophysiological changes in the monoaminergic system mimicking aspects of the schizophrenia phenotype in humans (10). HSV is also omnipresent in evolutionary history of man (11). However, all these characteristics of the virus make it a candidate, may give a hint toward causation, or provide a little circumstantial evidence, but are by no means proof of causation.

## The Hypothesis

Schizophrenia is a neurodevelopmental disease, with changes in the circuitry of the hippocampus shown postmortem, including a loss of lateralization in the dentate gyrus (12). The dentate gyrus is central to the cognitive adaptation of the human species across the life span, because this region is subject to adult neurogenesis, which secures life-long adaptation of memory to environmental influences, and neurogenesis is compromised in schizophrenia (13). The dentate gyrus is an primary target for HSV (14).

The virus hypothesis specified in this article is based on two remarkable phenomena, maybe combining to a third:

1. It is remarkable how pluripotent stem cells within the subgranular zone divide under influence of epigenetic mechanisms that (temporarily) silence the neuronal phenotype in favor of the stem cell phenotype, and how the suppression of neuronal phenotype is gradually relieved during differentiation into neurons (15). The master regulator of stemness and consecutive neuronal speciation is the transcription factor RE1 silencing transcription factor (REST) (16). REST is highly expressed in the milieu of the stem cell niche, suppresses hundreds of genes that are neuron specific, and thereby forces the cells in a dedifferentiated state, to enable replication. The niche, in which these stem cells replicate and form primitive neuronal progenitors, is the subgranular zone, and further differentiation into neurons occurs while they move toward the granular zone. This process of gradual speciation into neurons is guided by growth factors such as BDNF and nerve growth factor (NGF), which gradually suppress the transcription factor REST to enable neuronal genes to become more and more expressed on the way (17).
2. It is remarkable how in transneuronal tracing experiments the large DNA virus HSV, travels easily from the afferents of a sensory neuron deeply into the brain, without creating a severe encephalitis and death (18). Neuronal tracing experiments, with HSV, or with the related virus pseudo rabies virus (PRV), witness this peculiarity of nature. HSV travels anterogradely (along the normal direction of conduction of electrical stimuli along the axon), from the oral cavity sensory axon of the trigeminal nerve (Nervus V) via the efferent of this nerve to the next neuronal soma in the trigeminal nucleus (medulla oblongata), sending an efferent to the soma in the dopaminergic nuclei in the midbrain (substantia nigra), sending again an efferent into the limbic brain. An animal model shows (part) of this potential route of infection (19). The presence of HSV DNA as observed in the brain of healthy controls (20) is remarkable, because no apparent destruction is found on the supposed ways of anterograde transport of the virus (8). The suppression of replication of viral DNA in the infected neuron relies heavily on REST. Dominance of REST silences promoters of genes essential for replication of HSV and thereby prevents reactivation of the virus in neurons (21).
3. The remarkable hypothesis that might follow from the abovementioned is that stem cells in the dentate gyrus, which conserve stemness under influence of REST, contain very specific HSV gene elements that are forwarded into progenitors and thereby influence consecutive differentiation in a peculiar (schizophrenic) way: The necessary relief of REST to enable differentiation of the cell leads to partial derepression of the viral DNA element contained in the cell, which in combination with adverse life events (extreme social stress) is responsible for the neuroanatomical, neurophysiological, and behavioral phenotype of schizophrenia. The viral element present in stem cells, forwarded into progenitors, and partially expressed in differentiating cells, creates a certain variability on the differentiation process, that is not necessarily detrimental, but also might give rise to exceptional creativity, when kept in check. To be kept in check, the differentiation needs to be guided by growth factors that have a dual role: (1) to keep the virus in a sufficiently suppressed state to prevent activation of the (viral) replication machinery and (2) to support the developing neuron and its afferents with sufficient force to differentiate (cholinergic) transmitter connectivity that is important for new cognitions. NGF is produced within the hippocampus (22), supports cholinergic input to the hippocampus (23), and suppresses HSV (24).

## The Test

Some aspects of the hypothesis have been already proven in experiments: As mentioned above, in neuronal tracing experiments have been shown how easy HSV (or PRV) reaches limbic regions of the brain and how this in line with the dopamine hypothesis of schizophrenia. The connectivity of substantia nigra with the dentate gyrus of the hippocampus supports the possibility of anterograde transport of the virus to the neurogenetic niche (25). In herpes simplex virus infected progenitors, or immortalized monoaminergic PC12 cells the virus is kept latent by NGF signaling (26), but NGF also forces the primitive cells into a neuronal differentiation program (27). The major weakness of the current virus hypothesis is that the exact nature of the viral elements that supposedly interfere with the differentiation program in neuronal progenitors is not yet defined in detail. Many viral elements of HSV are kept latent by epigenetic silencing of promoters of these elements by REST. REST recruits histone deacetylases and lysine-specific demethylases (LSD) for suppression of gene transcription by epigenetic modification (28). When suppression by REST is relieved, which is observed during differentiation of the stem cell progenitor toward neuronal speciation, the activation of viral genes would occur in a predictive manner as observed in the earliest phase in very delicate viral reactivation experiments: When immediate early genes of the virus become relieved from suppression by REST, the genes essential for DNA replication genes of the virus [thymidine kinase (TK) or ribonucleotide reductase] become transcribed (29, 30) An important candidate viral element present in human brain is the TK gene of HSV (31), The HSV-TK gene contains a promoter with a Sp1 responsive element (32), which is activated due to stress signals and plays an important role in reactivation of the virus (33) and is involved in schizophrenia (34). In our hypothesis, the HSV-TK gene element may become activated upon stress and thereby disrupt neuronal speciation in the hippocampus and cause schizophrenia. For this specific hypothesis, all postulates of Koch regarding this specific viral gene/promoter have to apply. The viral element should be present in the stem cell niche of the patient, should interfere with neuronal speciation and integration of the neuron in the hippocampus under stress, and a similar pathophysiology has to apply when the viral element is introduced in the hippocampal stem cell niche in an animal model. These requirements could be fulfilled as follows: distract DNA from postmortem dentate gyrus brain material of schizophrenia patients, assuming that the HSV-TK gene is still present as in postmortem cases of herpes encephalitis (31). Amplify the DNA sequence found in the autopsy material containing both the enhancer/promoter binding site and the gene of interest (in our case HSV-TK). If amplified DNA copies of this viral element are found (only) in patient brain tissue, this will satisfy the first postulate. Then introduce the viral element discovered in human brain tissue, by knock-in into human NSC (neuronal stem cells) or PC12 cells that differentiate under control of NGF. Perform a detailed assessment of the phenotype of the differentiating cells with positive and negative control on the Sp1 stress response elements. If viral transduced neurons show a disrupted execution of the differentiation program and distrophical growth under Sp1 stress, the second postulate is fulfilled *in vitro*. Then, genetically introducing the viral element into mice hippocampi should provide us with the proof of the final postulate of Koch, the fact that the viral element transmits disease *in vivo*. In these experiments, the influence of the specific viral element discovered in human brain can be assessed regarding its influence on genesis, differentiation, migration and integration of neurons within the limbic network. To this aim, immunohistochemistry and fluorescent microscopy are very helpful, techniques that show us the normal neurogenetic mechanisms of cognition and what may go wrong (15).

## The Cure

Superficially, the cure for silencing of viral elements might seem easy: The only thing to do to keep the virus silent is suppress the transcription by silencing the promoter region of the viral element of interest epigenetically: keeping the activity of REST sufficiently high, as shown to be effective in viral knock-in REST gene experiments, would suffice (21). However, in patients, this pharmacological way to improve suppression by REST needs stepping out of the usual antipsychotic therapy of schizophrenia. We would need trials with drugs that support the epigenetically silencing REST complex, such as the LSD inhibitor Tranylcypromine (35). Is it a coincidence that LSD inhibitors are by another mechanism—by mono amino-oxidase inhibition—the most potent drugs to treat therapy resistant depression? Would suppression of viral elements by Tranylcypromine provide us with a cure for schizophrenia? Tranylcypromine might not only suppress expression of viral elements from HSV, but might also help to suppress other (retro)viral elements involved (4, 5). Suppressing the influence of stress on the expression of viral elements, might secure that neurogenesis is followed by proper differentiation of cell to neurons well integrated in the hippocampal circuitry and thereby prevent the accumulation of neurodevelopmental changes, found in postmortem hippocampi. It might basically cure schizophrenia by saving the patient from neurodevelopmental disarray. Medicinal treatment to *prevent* HSV replication might prove a far more effective treatment when compared with drugs that only *limit* HSV replication (36).

## Author Contributions

The author confirms being the sole contributor of this work and approved it for publication.

## Conflict of Interest Statement

The author declares that the research was conducted in the absence of any commercial or financial relationships that could be construed as a potential conflict of interest.
